# Is the Tradeoff between Folic Acid or/and Multivitamin Supplementation against Birth Defects in Early Pregnancy Reconsidered? Evidence Based on a Chinese Birth Cohort Study

**DOI:** 10.3390/nu15020279

**Published:** 2023-01-05

**Authors:** Jian Su, Shen Gao, Ruohua Yan, Ruixia Liu, Shaofei Su, Xiaolu Nie, Xiaohang Liu, Enjie Zhang, Shuanghua Xie, Jianhui Liu, Yue Zhang, Wentao Yue, Chenghong Yin, Xiaoxia Peng

**Affiliations:** 1 Center for Clinical Epidemiology and Evidence-based Medicine, Beijing Children’s Hospital, Capital Medical University, National Center for Children Health, Beijing 100045, China; 2 Department of Central Laboratory, Beijing Obstetrics and Gynecology Hospital, Capital Medical University, Beijing Maternal and Child Health Care Hospital, Beijing 100045, China; 3 Department of Research Management, Beijing Obstetrics and Gynecology Hospital, Capital Medical University, Beijing Maternal and Child Health Care Hospital, Beijing 100045, China

**Keywords:** birth defects, folic acid, multivitamin, two-level mixed-effect log binomial regression analysis

## Abstract

Background: Several studies have reported conflicting results on the association between maternal exposure to folic acid (FA) and/or multivitamin (MV) supplements and the risk of birth defects (BDs), especially for different subtypes of BDs. The present study aimed to identify the association between maternal exposure to FA or/and MV and BDs in offspring. Methods: In the Chinese Birth Cohort Study initiated from 20 November 2017, 120,652 pregnant women completed follow-up until 20 August 2021. The participants were classified into four groups: without exposure to FA and MV, exposure to only FA, exposure to only MV, and exposure to FA and MV. Birth defects were coded by the International Classification of Diseases (ICD)-10. In order to explore the structural relationship between maternal FA or MV supplements and BDs, directed acyclic graphs were drawn. Then, an inverse probability treatment weighting was utilized to reduce the systematic differences in the baseline characteristics among the different groups. Lastly, a two-level mixed-effect log binomial regression analysis was used to estimate the relative risk (RR) value of the different subtypes of BDs under different exposures to FA and/or MV. Results: Compared with the maternal group without exposure to FA and MV, the RR values of nervous system defects, face, ear, and neck defects, limb defects, and CHDs in the maternal group with only FA supplementation were less than 1.0, but they were not statistically significant. The RR values of genitourinary defects, abnormal chromosomes, and oral clefts were more than 1.0, and they were also not statistically significant. However, the risk of genitourinary defects (RR: 3.22, 95% CI: 1.42–7.29) and chromosomal abnormalities (RR: 2.57, 95% CI: 1.16–5.73) in the maternal group with only MV supplementation increased more than those in the maternal group without exposure to FA and MV. In addition, the RR values of all subtypes of BDs in the maternal group with exposure to FA and MV were closer to 1.0 than those in maternal group with exposure to only MV, but they were not statistically significant. Conclusions: It was indicated that the simultaneous supplementation of FA and MV in early pregnancy may have an interaction for the prevention of BDs and may have inconsistent effects for different subtypes of BDs. At the same time, excessive FA supplementation in pregnant women may increase the risk of BDs in their offspring. Although the mechanism is not clear, this evidence reminded us that more trade-offs are necessary for formulating strategies for the prevention of BDs with FA and/or MV supplementation in early pregnancy.

## 1. Introduction

Birth defects (BDs) or congenital abnormalities are defined as a series of structural, functional, or metabolic disorders that occur during embryonic or fetal development [1]. BDs can lead to premature birth, fetal death, infant death, and child disability and have become a global public health problem [2]. In 2015, the global disease burden ranked BDs as the fifth cause of death worldwide in children under the age of five. It was estimated that the incidence rate of BDs is higher in low-income and developing countries than in developed countries, and comprised 64.2, 55.7, and 47.2 per 1000 live births, respectively [3]. According to the surveillance of BDs in China, 5.6% of total newborns are born with BDs annually, which is equivalent to the level of developing countries. At present, one of the main causes of death in children aged 0–5 in China is BDs; about 45,000 children die of BDs every year [4]. Obviously, the burden of life loss caused by BDs is severe.

Micronutrient requirements increase significantly throughout pregnancy and are particularly important in the early stages of pregnancy when the infant’s major organs are formed. Studies have shown that the maternal nutritional status affects the growth and development of the fetus [5]. Folic acid (FA) is one of the most common supplements among women of reproductive age. Since two randomized controlled studies demonstrated that the maternal intake of FA supplements can prevent the occurrence of neural tube defects (NTDs) in the early 1990s [6,7], several countries, including the United States and Canada, have implemented mandatory FA supplementation in cereals as a public measure to prevent NTDs. In China, there is no mandatory FA fortification, but a nationwide public health project has been launched since 2009 recommending that women planning for pregnancy take 0.4 mg FA tablets daily from 3 months before pregnancy until 12 weeks of gestation [8]. However, several studies have demonstrated conflicting results on the associations between FA supplements and the risks of other types of BDs such as congenital heart defects (CHD), limb reduction defects, and cleft palates (CP) [9,10,11,12].

In addition, multivitamin (MV) supplementation is also an option for pregnant women during pregnancy in the Chinese consensus on clinically rational FA supplementation [13]. Elevit, the most commonly used MV in China, contains FA (0.8 mg) along with vitamin A (1.2 mg), vitamin D (12.5 μg), iron (60 mg), and zinc (7.5 mg). Several studies have shown that the occurrence of obstetric complications and offspring BDs are associated with vitamin deficiencies during pregnancy [14,15,16]. However, several studies have shown that an overdose of vitamins, such as the vitamin A contained in MVs, may increase the risk of BDs in offspring [17].

To our knowledge, evidence of the impacts of FA and/or MV supplements on the subtypes of BDs is limited worldwide. Therefore, a comprehensive analysis of the risks of organ-specific major BDs in the live and stillborn infants of mothers with different exposures to FA and/or MV supplements was conducted based on the China Birth Cohort Study (CBCS), which raised evidence for a further tradeoff between FA and MV supplements for the prevention of BDs.

## 2. Methods

### 2.1. Participants

The CBCS was a prospective, longitudinal, national-based birth cohort study [18], which was initiated in November 2017. The study was approved by the hospital ethics committee (approval number: 2018-KY-003-02), and all study subjects signed an informed consent form.

The women who were pregnant with 6 to 13 weeks + 6 days of gestation were recruited from 28 hospitals for gynecology and obstetrics nationwide. All eligible pregnant women followed up through an in-person interview based on a questionnaire at 20 to 23 weeks + 6 days, 28 to 33 weeks + 6 days of gestation. Corresponding clinical laboratory measures were collected at both of these follow-up visits, and the third follow up visit took place after delivery. If a participant miscarried in the first trimester, or miscarried in mid or late pregnancy, all clinical information was recorded by trained researchers, doctors, or nurses. If a BD was identified at any of these stages, clinical information, including ultrasound scan information on fetal defects, wase recorded, and biospecimens were collected by specially trained researchers, doctors, or nurses according to standard operating procedures and protocols [18].

**Figure 1:**
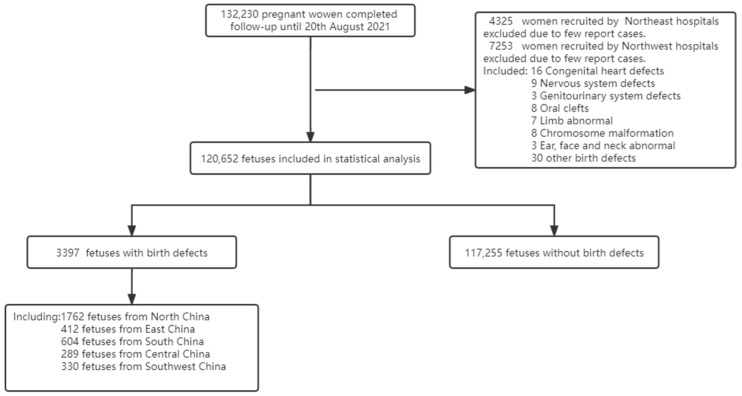
Flow chart of participants.

Until 20 August 2021, 132,230 pregnant women completed follow-up in the CBCS. After all the organ-specific major BDs in live and stillborn infants were confirmed by senior obstetricians, data cleaning was conducted. In addition, participants from several hospitals with a potential risk of selection bias were also excluded from the statistical analysis. Lastly, 120,652 participants were included (Figure 1).

When pregnant women in the first trimester of the gestational period were enrolled, they were asked questions, i.e., whether they had taken FA before pregnancy (yes or no); whether they had taken FA after pregnancy (yes or no); whether they had taken MV supplementation after pregnancy (yes or no). If they answered yes, they were asked to provide the brand name of the supplements. There is only one FA tablet available over the counter for pregnant women in China, which contained 0.4 mg of FA [19]. The most commonly used MV on the market in China contains 0.8 mg FA, vitamin A (1.2 mg), vitamin D (12.5 μg), iron (60 mg), and zinc (7.5 mg). Therefore, the exposure groups were classified as follows: without exposure to FA and MV, exposure to only FA, exposure to only MV, and exposure to FA and MV.

### 2.2. Classification of Birth Defects

All the confirmed BDs were coded according to the International Classification of Diseases (ICD)-10 [20]. The organ-specific major BDs were defined based on the following seven categories, including nervous system, ear–face–neck, congenital heart disease, oral clefts, genitourinary, limb, and chromosomal abnormality.

### 2.3. Statistical Analysis

Firstly, the baseline characteristics among four exposure groups of FA and/or MV were tested using a chi-square test or Fisher’s exact test (when conditions for the chi-square test were not met) for categorical variables, or Analysis of Variance (ANOVA) for continuous variables with approximate normal distribution. 

Secondly, the propensity score (PS) model was established to adjust the potential measured confounding and improve the balance between different exposure groups of FA and/or MV. The propensity score model was used instead of traditional multivariable statistical models based on two considerations: (1) the number of birth defects in some exposure groups was limited, rendering them unsuitable for statistical adjustment analysis due to several confounding factors; (2) the propensity score model has higher test efficiency in dealing with data with more confounding factors, especially for the lower incidence of outcome variables [21]. To fully understand the potential causal relationship between exposures, outcomes, and covariates [22], the directed acyclic graphs (DAGs) were configured to explore the structural relationship between maternal FA or MV supplements and BDs, referring to our previously published umbrella review, which comprehensively reviews the risk factors for CHDs [23]. The DAGitty in R software was used to construct the DAG, in which the variables on the causal path in DAG were included in the PS modeling as the covariates.

Inverse probability of treatment weighting (IPTW) was used to reduce systematic differences of baseline characteristics among participants exposed to FA or/and MV induced by covariates presented in the DAG figure [24]. Then, we calculated the inverse probability of the exposure weight of each participant, considering FA and/or MV according to PS. The PS weights were defined as 1/PS for participants exposed to FA or/and MV and 1/(1-PS) for participants exposed to neither FA nor MV. A standardized difference of less than 0.1 indicates that covariate imbalance between groups can be ignored [25]. In addition, extremely large weight values were not detected in IPTW distributions; therefore, we used all weights generated by the PS model for our analysis (Supplementary Figure S1).

It is well known that there is diversity in the diet, culture, and economic level among different regions of China. For example, the climate in South China is hot and rainy, plants grow luxuriantly, and there are many green leafy vegetables in the diet. East China is the most economically and culturally developed region, with rich water resources and relatively rich food types. The climate in North China is relatively cold, and the precipitation ensures that the consumption of green leafy vegetables is low. Southwest China has a bad climate, a complex geographical environment, and a relative shortage of food varieties. Considering that the above regional factors may affect the blood folate levels among pregnant women across different regions [26], a two-level mixed-effect log binomial regression analysis was used in this study because it had the potential to violate the principle of independence between participants when the correlation with the regional level was ignored, which could result in biased parameter estimates and will generally lead to underestimation of the standard errors and, accordingly, to incorrect conclusions about effect sizes (Supplementary Table S1) [27,28].

Lastly, crude (cRR) and adjusted risk ratios (aRR) and their 95% confidence intervals (CIs) were used to estimate the association between maternal exposure FA or/ and MV and BDs. All statistical tests were two sided, with a threshold for significance of p < 0.05. All analyses were performed using R software version 4.1.2.

## 3. Results

**Figure 2:**
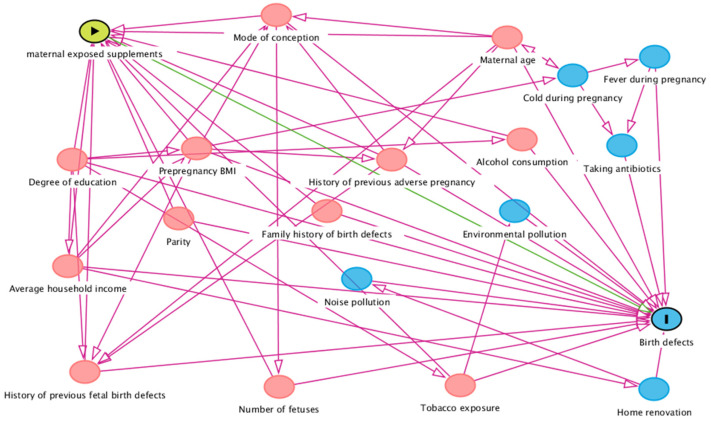
Directed acyclic graph based on supplements exposure and birth defects outcomes. Red circles represent variables included in the present statistical analysis and blue circles represent variables not included in the present statistical analysis. Right arrow in the yellow circle represent exposure factors, the vertical line in the blue circle represent outcome variable, the green line represent direct causal path.

Of all participants, which came to a total of 120,652 individuals, 40,204, 5567, 71,538, and 3343 pregnant women were exposed to only a FA supplement, only a MV supplement, both FA and MV, or neither FA nor MV, respectively. The baseline characteristics of participants from different exposure groups were shown in Table 1. The DAG figure was shown in Figure 2. Covariates on the DAG causal pathway include maternal age, maternal education, parity, family history of birth defects, pre-pregnancy body mass index (BMI), alcohol consumption during pregnancy, annual household income, history of previous adverse pregnancy, history of previous fetal birth defects, number of fetuses in this pregnancy, mode of conception, and tobacco exposure. The characteristics of the covariates demonstrated in the DAG figure among four groups were not comparable before applying IPTW, in which standardized differences among groups were more than 0.1. The standardized difference among groups was less than 0.1 after IPTW.

The incidence of BDs in four exposure groups were shown in Table 2. The CHDs were the most common subtype of BDs (70.3/10,000), followed by abnormal chromosome (42.9/10,000), the genitourinary system (42.2/10,000), nervous system (36.2/10,000), limb (31.2/10,000), ear, face and neck (30.6/10,000), and oral clefts (14.4/10,000). No matter the subtype of BDs, the incidence of BDs in the exposure group with FA and MV was higher than that in other groups. The incidence of BDs in five regions of China was shown in Table 3, showing regional differences in BDs incidence. The incidence of BDs in north China was higher than in other regions.

The analysis results based on the two-level mixed-effect log binomial regression analysis were shown in Table 4. Compared to mothers exposed to neither FA nor MV (i.e., the reference group), the RR values of nervous system defects; face, ear, and neck defects; limb defects; and CHDs in the maternal group taking only the FA supplement were less than 1.0, but not statistically significant. The RR values of genitourinary defects, abnormal chromosome, and oral clefts were more than 1.0, which was also not statistically significant. In addition, the RR values in the maternal group taking only the MV supplement were less than 1.0 but were not statistically significant, including nervous system defects and face, ear, and neck defects. The RR values of CHDs, limb defects, and oral clefts were more than 1.0, which was also not statistically significant. The risk of genitourinary defect and abnormal chromosomal in maternal group with only MV supplement increased inconsistently compared to those in the maternal group without Exposure to FA and MV. It is noteworthy that the RR of genitourinary defect and abnormal chromosomal in the maternal group with exposure to FA and MV decreased compared to that in the maternal group taking only the MV supplement.

## 4. Discussion

This study was based on the current largest birth cohort in China. Thirty-three hundred and ninety infants with BDs were diagnosed from 120,652 participants, covering live births, stillbirths, and pregnancy terminations after the prenatal diagnosis of any BDs at any gestational age. The impacts of maternal FA or/and MV supplements on various subtypes of BDs were shown to be different, including the direction of associations and effect sizes.

It is known that FA could prevent the occurrence of NTDs [29]. With the implementation of the FA supplementation policy in China, NTDs was no longer the leading cause of BDs [30]. This has led to interest in the effect of FA supplementation on other subtypes of BDs. On the other hand, a research hotspot, i.e., whether a higher FA dose could effectively prevent BDs, has also arisen in recent years. For example, a preconception cohort conducted in Shanghai, China showed that higher maternal red blood cell (RBC) folate was associated with reduced offspring risk of CHDs [31].

The present study analyzed the impacts of FA and/or MV supplementations on different types of BDs. Consistent with the prospective study conducted based on population-based Medical Birth Registry of Norway (MBRN) from 1999–2013 [32], the results based on the Chinese birth cohort also showed that there were associational disparities between FA supplementation and the subtype of BDs. For example, the risk of CHDs; nervous system defects; ear, face, and neck defects; and limb defects seemed to decrease following FA supplementation, but the risk of genitourinary, chromosome, and oral clefts defects seemed to increase (Table 4). However, it should be noted that the number of genitourinary and chromosome defects was below ten in the group without exposure to FA and MV, which may mean that the lower CIs of RR in group with exposure to FA and MV for genitourinary and chromosomal defects would be greater than 1.0 with the light increase in BDs cases. These findings pointed out that we should pay attention to the effect of disparities of FA on different subtypes of BDs in offspring.

Exceeding our expectations, maternal exposure to only MV, which contains not only higher doses of FA (0.8 mg), but also vitamin A (1.2 mg), vitamin D (12.5 μg), iron (60 mg), and zinc (7.5 mg), was associated with an increased risk of offspring genitourinary defects and abnormal chromosomes with statistical significance. A potential explanation was that the excessive accumulation of fat-soluble vitamins from MV could increase the risk of some subtypes of BDs, which had been reported recently [32]. The MV products sold in China contain vitamin A, which was easy to accumulate in the body and reached a higher concentration [33]. Exposure to high doses of vitamin A might affect fetal palatogenesis by interfering with cell proliferation, and was also found in animal studies of neural tube closure and organ and limb development [17,34]. Although the vitamin A level of maternal exposure in the present study was not measured, which limited further study on the causality between MV and BDs, the recommendations of mothers in early pregnancy to use MV should be treated with caution. 

Another possible reason for this may be the potential negative effect of FA on zinc absorption in MV [35,36]. Zinc was closely associated with the synthesis of a variety of enzymes, nucleic acids, and proteins, and has an extremely important effect on embryonic growth and development. Therefore, maternal zinc deficiency may cause fetal NTDs, low birth weight, and intrauterine growth retardation [37,38]. One study showed that plasma zinc levels were negatively correlated with levels of FA in pregnant women [39]. To determine the mechanism by which FA and zinc interact in the intestine, transport studies in vivo and in vitro were performed [40], which showed that in the intestinal lumen, transport of zinc decreased significantly when FA was present and vice versa. Bidirectional inhibition of zinc and folate transport in the intestine occurred under normal physiological conditions, and under acidic conditions in the stomach, zine and folate could form poorly soluble complexes, thus affecting the mutual metabolic absorption. In our study, when FA and MV are supplemented simultaneously in the first trimester of pregnancy, FA may interact with zinc in MV and demonstrate inconsistent effects for different subtypes of BDs.

Several studies have shown that higher maternal RBC folate was associated with reduced certain subtypes of BDs, such as CHDs and NTDs, in offspring [31]. However, they did not take into account the influence regarding the adverse effects of high folate levels or non-absorption of folate on the mother and fetus.

At present, the American institute of medicine and the European food safety agency recommend that the upper intake level (UL) of FA is 1000 μg. Excessive intake of FA may lead to an increase in unmetabolized folic acid (UMFA) in the bloodstream. It was found that, unlike natural folates, synthetic folate must first be reduced to tetrahydrofolate by dihydrofolate reductase (DHFR) and then methylated to 5- methylenetetrahydrofolate (5-MTHF) by methylenetetrahydrofolate reductase (MTHFR). Unlike mammals, DHFR activity in humans is low and varies widely among individuals [41]. Showing efficacy at doses over 200–400 μg, folate uptake and bio metabolic capacity then reach saturation [42]. The limitations of this metabolic process result in an inability to metabolize high doses of folate, leading to the appearance of UMFA in the circulation. Intake of more than 1 mg of FA per day can significantly increase UMFA in serum, and the increased UMFA can inhibit DHFR activity in liver, further weakening FA metabolism and clearance, leading to accumulation in the body [43]. In our study, FA in the daily diet of pregnant women plus FA and MV supplements were likely to exceed UL, which would lead to the appearance of UMFA in the body and thus affect the metabolism of FA. Concerning the possible deleterious effects of excess folic acid supplementation, a group of experts gathered by the NIH have pointed out that there is an insufficient body of evidence to support human adverse health outcomes that are a result of high amounts of FA intake, although pregnant women and their children are at high risk of FA overexposure. [44]. On the other hand, some studies have shown that maternal exposure to excessive FA may lead to autism spectrum disorder [45,46], asthma [47], and metabolic abnormalities [48] in offspring. The present studies may provide evidence of the deleterious effects of maternal excessive folate intake on the potentially increased risk of BDs in offspring, especially for some subtypes of BDs.

Another reason may be that higher maternal RBC folate level in early pregnancy was significantly associated with Gestational Diabetes Mellitus (GDM) risk [49,50,51], and maternal GDM was a risk factor for BDs in offspring [23,52]. A prospective study from China [50] has shown that daily FA supplementation in early pregnancy increases the risk of GDM (OR 1.73, 95% CI 1.19–2.53). Compared with RBC folate <400 ng/mL, pregnancies with RBC folate ≥600 ng/mL were associated with about 1.60-fold higher odds of GDM (OR 1.58, 95% CI 1.03–2.41). At the same time, our study also showed that the incidence of GDM in participants was positively correlated with folate dose (Supplementary Table S2).

Our study has several strengths. First, CBCS provides good data support for our research. CBCS is a prospective design with large sample size, and the research protocol has been published. Strict data quality control and accurate diagnosis of BDs can ensure the authenticity and reliability. Secondly, we apply DAG to visualize the complex causal relationships between maternal supplements exposure and offspring BDs, which can provide an intuitive method for identifying confounding, and transformation of identifying confounding into minimally sufficient adjustment sets. We also use IPTW to account for systematic differences in selected DAG covariates, which allowed measured baseline covariates between groups to become comparable after weighting. Considering the regional clustering of participants, we performed multilevel modeling; this helps to explain the fixed effects of both the individual and region-level factors and the random intercept to explain the between-regional cluster differences concurrently. This was a third strength of our study.

However, we noticed some limitations of our study. First, our study was qualitative, which limited us to quantitative exploration of the dose–response relationship between maternal FA exposure in early pregnancy and subtypes of BDs in offspring. Second, the referral hospitals in our study were almost 3A hospitals. Thus, Berkson bias may be apparent due to different admission rates or different access opportunities [53]. Additionally, most of the participants were from cities; compared with people living in rural areas, they may have better living habits and health awareness, and better compliance with supplements use. Although we used IPTW to balance various measurable confounders of maternal supplement exposure, there is still some unmeasurable and residual confounding at play. Third, we did not collect FA doses from participants’ daily diets, which may affect our result. However, most of the participants were of Han ethnicity, and traditional regional diet patterns may play a role in the results. However, we took regional factors as a random effect using a two-level log binomial model; this would not have changed the overall trends observed in our study. Lastly, the potential adverse effects of trace elements on birth defects were indicated only based on an epidemiology survey and do not have appropriate biological assays to support this hypothesis. Subsequently, we will carry out corresponding experiments to verify this hypothesis in the future based on the biology specimen in CBCS.

In conclusion, simultaneous supplementation of FA and MV during the first trimester may have interactive effects on the prevention of BDs and inconsistent effects on different subtypes of BDs. At the same time, attention should be paid to the teratogenic effects of vitamin A and the adverse effects of excess FA intake. Although the mechanisms remain unclear, this evidence reminds us that additional trade-offs are needed when developing prevention strategies for BDs with FA and/or MV supplementation in the first trimester.

## Figures and Tables

**Table 1:** Demographics and characteristics of participants.

Characteristics (*n*, %)	Unweighted Cohort					Inverse Probability Weighted Cohort				
	Exposure to only FA (*n =* 40,204)	Exposure to only MV (*n =* 5567)	Exposure to FA and MV (*n =* 71,538)	Without Exposure to FA and MV (*n =* 3343)	Standardized Difference	Exposure to only FA	Exposure to only MV	Exposure to FA and MV	Without Exposure to FA and MV	Standardized Difference
Maternal age, *n* (%)					0.106					0.007
<35	37,026 (92.1)	4864 (87.4)	64,571 (90.3)	2892 (86.5)		90.5	90.2	90.6	90.4	
≥35	3178 (7.9)	703 (12.6)	6967 (9.7)	451 (13.5)		9.5	9.8	9.4	9.6	
Pre-pregnancy BMI, kg/m^2^, *n* (%)					0.075					0.019
<18.5	26,312 (65.4)	3752 (67.4)	48,004 (67.1)	2119 (63.4)		66.5	66.1	66.4	67	
18.5–24	5682 (14.1)	714 (12.8)	9242 (12.9)	431 (12.9)		13.3	13.9	13.3	13.4	
24–28	6165 (15.3)	823 (14.8)	10,904 (15.2)	544 (16.3)		15.4	15.2	15.3	14.6	
≥28	2045 (5.1)	278 (5.0)	3388 (4.7)	249 (7.4)		4.9	4.8	4.9	5.1	
Maternal education, *n* (%)					0.389					0.018
Primary school or lower	222 (0.6)	7 (0.1)	225 (0.3)	60 (1.8)		0.4	0.5	0.4	0.5	
High school or lower	11,400 (28.4)	628 (11.3)	11,617 (16.2)	1249 (37.4)		20.5	20.5	20.6	21.7	
Bachelor’s degree or above	28,582 (71.1)	4932 (88.6)	59,696 (83.4)	2034 (60.8)		79.1	79	79	77.8	
Family annually income, Yuan, *n* (%)										
<200 K	29,262(72.8)	2757 (49.5)	46,103 (64.4)	2569 (76.8)	0.323	66.6	66.7	66.8	69.2	0.062
≥200 K	10,942(27.2)	2810 (50.5)	25,435 (35.6)	774 (23.2)		33.4	33.3	33.2	30.8	
Parity, *n* (%)					0.311					0.012
0	18,202 (45.3)	2633 (47.3)	36,237 (50.7)	910 (27.2)		48.1	47.7	48	47.1	
1	20,053 (49.9)	2793 (50.2)	33,317 (46.6)	2014 (60.2)		48.2	48.5	48.2	48.9	
≥2	1949 (4.8)	141 (2.5)	1984 (2.8)	419 (12.5)		3.7	3.8	3.7	4	
Number of fetuses, *n* (%)					0.053					0.005
1	39,633 (98.6)	5412 (97.2)	69,933 (97.8)	3283 (98.2)		98	98	98	98.1	
≥2	571 (1.4)	155 (2.8)	1605 (2.2)	60 (1.8)		2	2	2	1.9	
Maternal alcohol drinking, *n* (%)					0.037					0.003
No	38,876 (96.7)	5323 (95.6)	69,361 (97.0)	3227 (96.5)		96.8	96.7	96.8	96.8	
Yes	1328 (3.3)	244 (4.4)	2177 (3.0)	116 (3.5)		3.2	3.3	3.2	3.2	
Maternal tobacco exposure, *n* (%)					0.095					0.006
No	34,956 (86.9)	4966 (89.2)	64,228 (89.8)	2815 (84.2)		88.8	88.5	88.7	88.5	
Yes	5248 (13.1)	601 (10.8)	7310 (10.2)	528 (15.8)		11.2	11.5	11.3	11.5	
Mode of conception, *n* (%)					0.213					0.015
Natural pregnancy	39,342 (97.9)	5093 (91.5)	67,111 (93.8)	3305 (98.9)		94.9	94.8	95.2	94.5	
Assisted reproduction	862 (2.1)	474 (8.5)	4427 (6.2)	38 (1.1)		5.1	5.2	4.8	5.5	
Family history of birth defects, *n* (%)					0.025					0.005
No	38,920 (96.8)	5340 (95.9)	69,247 (96.8)	3238 (96.9)		96.8	96.8	96.8	96.9	
Yes	1284 (3.2)	227 (4.1)	2291 (3.2)	105 (3.1)		3.2	3.2	3.2	3.1	
History of adverse pregnancy, *n* (%)					0.09					0.005
No	26,579 (66.1)	3597 (64.6)	49,036 (68.5)	2020 (60.4)		67.4	67.2	67.3	66.9	
Yes	13,625 (33.9)	1970 (35.4)	22,502 (31.5)	1323 (39.6)		32.6	32.8	32.7	33.1	
History of previous birth defect pregnancy, *n* (%)					0.014					0.008
No	39,443 (98.1)	5446 (97.8)	70,067 (97.9)	3282 (98.2)		98	98	98	98.2	
Yes	761 (1.9)	121 (2.2)	1471 (2.1)	61 (1.8)		2	2	2	1.8	

Abbreviations: FA—folic acid; MV—multivitamin. Data are presented as *n* (%).

**Table 2:** Incidence and its 95% confidence interval of major birth defects in different exposure group.

Birth Defects	Total (120,652)		Exposure to Only FA (40,204)		Exposure to Only MV (5567)		Exposure to FA and MV (71,538)		Without Exposure to FA and MV (3343)	
	*n*	Incidence per 10,000	*n*	Incidence per 10,000	*n*	Incidence per 10,000	*n*	Incidence per 10,000	*n*	Incidence per 10,000
Congenital heart defects	848	70.3 (65.6–75.2)	204	50.7 (44.0–58.2)	57	102.4 (77.6–132.5)	569	79.5 (73.2–86.3)	18	53.8 (31.9–85.0)
Abnormal chromosome	517	42.9 (39.2–46.7)	146	36.3 (30.7–42.7)	41	73.6 (52.9–99.8)	321	44.9 (40.1–50.0)	9	26.9 (12.3–51.0)
Genitourinary system	509	42.2 (38.6–46.0)	133	33.1 (27.7–39.2)	51	91.6 (68.3–120.3)	319	44.6 (39.4–49.8)	6	17.9 (6.6–39.0)
Nervous system	437	36.2 (32.9–39.8)	111	27.6 (22.7–33.2)	27	48.5 (32.0–70.4)	287	40.1 (35.6–45.0)	12	35.9 (18.6–62.6)
Limb	376	31.2 (28.1–34.5)	105	26.1 (21.4–31.6)	38	68.3 (48.3–93.6)	223	31.2 (27.2–35.5)	10	29.9 (14.4–54.9)
Ear, face and neck	369	30.6 (27.5–33.9)	103	25.6 (20.9–31.1)	32	57.5 (39.3–81.1)	225	31.5 (27.5–35.8)	9	26.9 (12.3–51.0)
Oral clefts	174	14.4 (12.4–16.7)	48	11.9 (8.8–15.8)	9	16.2 (7.4–30.7)	114	15.9 (13.1–19.1)	3	9.0 (1.8–26.2)

Abbreviations: FA, folic acid; MV, multivitamin.

**Table 3:** Incidence and its 95% confidence interval of major birth defects across different regions in China.

Birth Defects	North China (42,968)		South China (22,724)		East China (24,070)		Central China (13,351)		Southwest China (17,539)	
	*n*	Incidence per 10,000	*n*	Incidence per 10,000	*n*	Incidence per 10,000	*n*	Incidence per 10,000	*n*	Incidence per 10,000
Congenital heart defects	395	91.9 (83.1–101.4)	155	68.2 (57.9–79.8)	123	51.1 (42.5–60.9)	100	74.9 (61.0–91.0)	75	42.8 (33.6–53.6)
Abnormal chromosome	268	62.4 (55.1–70.3)	111	48.8 (40.2–58.8)	39	16.2 (11.5–22.1)	51	38.2 (28.5–50.2)	48	27.4 (20.2–36.3)
Genitourinary system	338	78.7 (70.5–87.5)	77	33.9 (26.8–42.3)	40	16.6 (11.9–22.6)	25	18.7 (12.1–27.6)	29	16.5 (11.1–23.7)
Nervous system	216	50.3 (43.8–57.4)	103	45.3 (37.0–54.9)	41	17.0 (12.2–23.1)	33	24.7 (17.0–34.7)	44	25.1 (18.2–33.7)
Limb	156	36.3 (30.8–42.5)	107	47.1 (38.6–56.9)	41	17.0 (12.2–23.1)	33	24.7 (17.0–34.7)	39	22.2 (15.8–30.4)
Ear, face, and neck	281	65.4 (58.0–73.5)	41	18.0 (13.0–24.5)	17	7.1 (4.1–11.3)	7	5.2 (2.1–10.8)	23	13.1 (8.3–19.7)
Oral clefts	74	17.2 (13.5–21.6)	27	11.9 (7.8–17.3)	33	13.7 (9.4–19.2)	16	12.0 (6.9–19.5)	24	13.7 (8.8–20.3)

**Table 4:** The adjusted RR and its 95% confidence interval of major birth defects in different exposure groups.

Diseases	Without Exposure to FA and MV	Exposure to Only FA	Exposure to Only MV	Exposure to FA and MV
Congenital heart disease	1 (Reference)	0.95 (0.60–1.51)	1.42 (0.84–2.39)	1.25 (0.79–1.96)
Chromosome	1 (Reference)	1.85 (0.87–3.90)	2.57 (1.16–5.73) *	1.93 (0.92–4.04)
Genitourinary	1 (Reference)	1.97 (0.90–4.30)	3.22 (1.42–7.29) *	1.98 (0.91–4.30)
Nervous system	1 (Reference)	0.75 (0.42–1.35)	0.86 (0.43–1.73)	0.95 (0.54–1.68)
Limb	1 (Reference)	0.93 (0.49–1.79)	1.97 (0.97–4.00)	0.97 (0.51–1.84)
Face, ear, and neck	1 (Reference)	0.84 (0.46–1.54)	0.97 (0.48–1.94)	0.76 (0.42–1.39)
Oral clefts	1 (Reference)	1.23 (0.38–4.00)	1.48 (0.38–5.67)	1.64 (0.52–5.23)

Abbreviations: FA—folic acid; MV—multivitamin. * the results are statistically significant.

## Data Availability

Not applicable.

## Supplementary Materials

The following supporting information can be downloaded at: https://www.mdpi.com/article/10.3390/nu15020279/s1, Figure S1: Distribution of weight generated by IPTW; Table S1: Two-level log-binominal model results, among different subtypes of birth defects; Table S2: Incidence of Gestational Diabetes Mellitus among different exposure groups.

## References

[1] Pai G.S., Gadewar S.B. (2000). Diagnostic approach to children with birth defects. Indian J. Pediatr..

[2] Tuan R.S. (2017). Birth Defects: Etiology, screening, and detection. Birth Defects Res..

[3] GBD 2015 Child Mortality Collaborators (2016). Global, regional, national, and selected subnational levels of stillbirths, neonatal, infant, and under-5 mortality, 1980–2015: A systematic analysis for the Global Burden of Disease Study 2015. Lancet.

[4] He C., Liu L., Chu Y., Perin J., Dai L., Li X., Miao L., Kang L., Li Q., Scherpbier R. (2017). National and subnational all-cause and cause-specific child mortality in China, 1996–2015: A systematic analysis with implications for the Sustainable Development Goals. Lancet Glob. Health.

[5] Gernand A.D., Schulze K.J., Stewart C.P., West K.P., Christian P. (2016). Micronutrient deficiencies in pregnancy worldwide: Health effects and prevention. Nat. Rev. Endocrinol..

[6] MRC Vitamin Study Research Group (1991). Prevention of neural tube defects: Results of the Medical Research Council Vitamin Study. Lancet.

[7] Czeizel A.E., Dudas I. (1992). Prevention of the first occurrence of neural-tube defects by periconceptional vitamin supplementation. N. Engl. J. Med..

[8] Liu J., Jin L., Meng Q., Gao L., Zhang L., Li Z., Ren A. (2015). Changes in folic acid supplementation behaviour among women of reproductive age after the implementation of a massive supplementation programme in China. Public Health Nutr..

[9] Oyen N., Olsen S.F., Basit S., Leirgul E., Strøm M., Carstensen L., Granström C., Tell G.S., Magnus P., Vollset S.E. (2019). Association Between Maternal Folic Acid Supplementation and Congenital Heart Defects in Offspring in Birth Cohorts from Denmark and Norway. J. Am. Heart Assoc..

[10] De-Regil L.M., Pena-Rosas J.P., Fernandez-Gaxiola A.C., Rayco-Solon P. (2015). Effects and safety of periconceptional oral folate supplementation for preventing birth defects. Cochrane Database Syst. Rev..

[11] Werler M.M., Hayes C., Louik C., Shapiro S., Mitchell A.A. (1999). Multivitamin supplementation and risk of birth defects. Am. J. Epidemiol..

[12] Botto L.D., Olney R.S., Erickson J.D. (2004). Vitamin supplements and the risk for congenital anomalies other than neural tube defects. Am. J. Med. Genet. C Semin. Med. Genet..

[13] Compilation expert group of “Multidisciplinary Expert Consensus on Rational Clinical Supplementation of Folic Acid in China” (2021). Multidisciplinary expert consensus on rational folic acid supplementation in China. Her. Med..

[14] Smedts H.P., de Vries J.H., Rakhshandehroo M., Wildhagen M.F., Verkleij-Hagoort A.C., Steegers E.A., Steegers-Theunissen R.P. (2009). High maternal vitamin E intake by diet or supplements is associated with congenital heart defects in the offspring. BJOG.

[15] Hanson C., Jones G., Lyden E., Kaufmann M., Armas L., Anderson-Berry A. (2016). Vitamin D metabolism in the premature newborn: A randomized trial. Clin. Nutr..

[16] Rumbold A., Ota E., Hori H., Miyazaki C., Crowther C.A. (2015). Vitamin E supplementation in pregnancy. Cochrane Database Syst. Rev..

[17] Ackermans M.M., Zhou H., Carels C.E., Wagener F.A., Von den Hoff J.W. (2011). Vitamin A and clefting: Putative biological mechanisms. Nutr. Rev..

[18] Yue W., Zhang E., Liu R., Zhang Y., Wang C., Gao S., Su S., Gao X., Wu Q., Yang X. (2022). The China birth cohort study (CBCS). Eur. J. Epidemiol..

[19] Zhang X., Liu J., Jin Y., Yang S., Song Z., Jin L., Wang L., Ren A. (2019). Folate of pregnant women after a nationwide folic acid supplementation in China. Matern. Child. Nutr..

[20] Hirsch J.A., Leslie-Mazwi T.M., Nicola G.N., Oklu R., Schoppe K.A., Silva E., Manchikanti L. (2015). The ICD-10 system: A gift that keeps on taking. J. Neurointerv. Surg..

[21] Haukoos J.S., Lewis R.J. (2015). The Propensity Score. JAMA.

[22] Yan R., Liu T., Peng Y. (2020). Can statistical adjustment guided by causal inference improve the accuracy of effect estimation? A simulation and empirical research based on meta-analyses of case-control studies. BMC Med. Inform. Decis. Mak..

[23] Nie X., Liu X., Wang C., Wu Z., Sun Z., Su J., Yan R., Peng Y., Yang Y., Wang C. (2022). Assessment of evidence on reported non-genetic risk factors of congenital heart defects: The updated umbrella review. BMC Pregnancy Childbirth.

[24] Austin P.C., Grootendorst P., Anderson G.M. (2007). A comparison of the ability of different propensity score models to balance measured variables between treated and untreated subjects: A Monte Carlo study. Stat. Med..

[25] Austin P.C., Stuart E.A. (2015). Moving towards best practice when using inverse probability of treatment weighting (IPTW) using the propensity score to estimate causal treatment effects in observational studies. Stat. Med..

[26] Ren A., Zhang L., Hao L., Li Z., Tian Y., Li Z. (2007). Comparison of blood folate levels among pregnant Chinese women in areas with high and low prevalence of neural tube defects. Public Health Nutr..

[27] Birhanu B.E., Kebede D.L., Kahsay A.B., Belachew A.B. (2019). Predictors of teenage pregnancy in Ethiopia: A multilevel analysis. BMC Public Health.

[28] Peugh J.L. (2010). A practical guide to multilevel modeling. J. Sch. Psychol..

[29] Dean J.H., Pauly R., Stevenson R.E. (2020). Neural Tube Defects and Associated Anomalies before and after Folic Acid Fortification. J. Pediatr..

[30] Dai L., Zhu J., Liang J., Wang Y.P., Wang H., Mao M. (2011). Birth defects surveillance in China. World J. Pediatr..

[31] Chen H., Zhang Y., Wang D., Chen X., Li M., Huang X., Jiang Y., Dou Y., Wang Y., Ma X. (2022). Periconception Red Blood Cell Folate and Offspring Congenital Heart Disease: Nested Case-Control and Mendelian Randomization Studies. Ann. Intern. Med..

[32] Gildestad T., Bjorge T., Haaland O.A., Klungsøyr K., Vollset S.E., Øyen N. (2020). Maternal use of folic acid and multivitamin supplements and infant risk of birth defects in Norway, 1999–2013. Br. J. Nutr..

[33] Martinez-Frias M.L., Salvador J. (1990). Epidemiological aspects of prenatal exposure to high doses of vitamin A in Spain. Eur. J. Epidemiol..

[34] Abbott B.D., Harris M.W., Birnbaum L.S. (1989). Etiology of retinoic acid-induced cleft palate varies with the embryonic stage. Teratology.

[35] Arnaud J., Favier A., Herrmann M.A., Pilorget J.J. (1992). [Effect of folic acid and folinic acid on zinc absorption]. Ann. Nutr. Metab..

[36] Milne D.B., Canfield W.K., Mahalko J.R., Sandstead H.H. (1984). Effect of oral folic acid supplements on zinc, copper, and iron absorption and excretion. Am. J. Clin. Nutr..

[37] Cherry F.F., Bennett E.A., Bazzano G.S., Johnson L.K., Fosmire G.J., Batson H.K. (1981). Plasma zinc in hypertension/toxemia and other reproductive variables in adolescent pregnancy. Am. J. Clin. Nutr..

[38] Simmer K., Thompson R.P. (1985). Maternal zinc and intrauterine growth retardation. Clin. Sci..

[39] Mukherjee M.D., Sandstead H.H., Ratnaparkhi M.V., Johnson L.K., Milne D.B., Stelling H.P. (1984). Maternal zinc, iron, folic acid, and protein nutriture and outcome of human pregnancy. Am. J. Clin. Nutr..

[40] Ghishan F.K., Said H.M., Wilson P.C., Murrell J.E., Greene H.L. (1986). Intestinal transport of zinc and folic acid: A mutual inhibitory effect. Am. J. Clin. Nutr..

[41] Pietrzik K., Bailey L., Shane B. (2010). Folic acid and L-5-methyltetrahydrofolate: Comparison of clinical pharmacokinetics and pharmacodynamics. Clin. Pharmacokinet..

[42] Sweeney M.R., McPartlin J., Scott J. (2007). Folic acid fortification and public health: Report on threshold doses above which unmetabolised folic acid appear in serum. BMC Public Health.

[43] Bailey S.W., Ayling J.E. (2009). The extremely slow and variable activity of dihydrofolate reductase in human liver and its implications for high folic acid intake. Proc. Natl. Acad. Sci. USA.

[44] Maruvada P., Stover P.J., Mason J.B., Bailey R.L., Davis C.D., Field M.S., Finnell R.H., Garza C., Green R., Gueant J.L. (2020). Knowledge gaps in understanding the metabolic and clinical effects of ex-cess folates/folic acid: A summary, and perspectives, from an NIH workshop. Am. J. Clin. Nutr..

[45] Valera-Gran D., de la Hera M.G., Navarrete-Munoz E.M., Fernandez-Somoano A., Tardón A., Julvez J., Forns J., Lertxundi N., Ibarluzea J.M., Murcia M. (2014). Folic acid supplements during pregnancy and child psychomotor development after the first year of life. JAMA Pediatr..

[46] Shorter K.R., Felder M.R., Vrana P.B. (2015). Consequences of dietary methyl donor supplements: Is more always better?. Prog. Biophys. Mol. Biol..

[47] Silva C., Keating E., Pinto E. (2017). The impact of folic acid supplementation on gestational and long term health: Critical temporal windows, benefits and risks. Porto Biomed. J..

[48] Yajnik C.S., Deshpande S.S., Jackson A.A., Refsum H., Rao S., Fisher D.J., Bhat D.S., Naik S.S., Coyaji K.J., Joglekar C.V. (2008). Vitamin B12 and folate concentrations during pregnancy and insulin resistance in the offspring: The Pune Maternal Nutrition Study. Diabetologia.

[49] Xie K., Xu P., Fu Z., Gu X., Li H., Cui X., You L., Zhu L., Ji C., Guo X. (2019). Association of maternal folate status in the second trimester of pregnancy with the risk of gestational diabetes mellitus. Food Sci. Nutr..

[50] Chen X., Zhang Y., Chen H., Jiang Y., Wang Y., Wang D., Li M., Dou Y., Sun X., Huang G. (2021). Association of Maternal Folate and Vitamin B12 in Early Pregnancy With Gestational Diabetes Mellitus: A Prospective Cohort Study. Diabetes Care.

[51] Li S., Hou Y., Yan X., Wang Y., Shi C., Wu X., Liu H., Zhang L., Zhang X., Liu J. (2019). Joint effects of folate and vitamin B12 imbalance with maternal characteristics on gestational diabetes mellitus. J. Diabetes.

[52] Wu Y., Liu B., Sun Y., Du Y., Santillan M.K., Santillan D.A., Snetselaar L.G., Bao W. (2020). Association of Maternal Prepregnancy Diabetes and Gestational Diabetes Mellitus With Congenital Anomalies of the Newborn. Diabetes Care.

[53] Sadetzki S., Bensal D., Novikov I., Modan B. (2003). The limitations of using hospital controls in cancer etiology--one more ex-ample for Berkson’s bias. Eur. J. Epidemiol..

