# Supplementary material for: Is the Tradeoff between Folic Acid or/and Multivitamin Supplementation against Birth Defects in Early Pregnancy Reconsidered? Evidence Based on a Chinese Birth Cohort Study
Source: Nutrients. 2023 Jan 5;15(2):279. doi: 10.3390/nu15020279 (PMC9865336; doi:10.3390/nu15020279)
Supplement: Supplementary file 1 [file nutrients-15-00279-s001.zip › Table S1.pdf]

Table S1. Two level log-binominal model results among different subtypes of birth defects.

Birth defects ICC= 2.99%

**Covariates**	**Estimated**	**Standard error**	**Odds ratio（95%CI）**	**T-value**	**P-value**
Fixed effects					
Intercept	-3.80	0.18		-21.33	<.0001
**Take supplements**					
None	Ref	Ref	Ref	Ref	Ref
Taking folic acid only	0.01	0.12	1.01(0.81-1.28)	1.01	0.91
Taking multivitamin only	0.36	0.13	1.44(1.11-1.86)	1.44	0.01
Taking folic acid plus multivitamins	0.12	0.12	1.13(0.90-1.42)	1.13	0.29
**Random effects**					
Intercept	0.10	0.31		0.32	0.38

Congenital heart defects ICC= 2.13%

**Covariates**	**Estimated**	**Standard error**	**Odds ratio (95%CI)**	**T-value**	**P-value**
**Fixed effects**					
Intercept	-5.14	0.25		-20.47	<.0001
Take supplements					
None	Ref	Ref	Ref	Ref	Ref
Taking folic acid only	-0.05	0.24	0.98(0.58-1.64)	-0.20	0.84
Taking multivitamin only	0.35	0.27	1.59(0.89-2.83)	1.32	0.19
Taking folic acid plus multivitamins	0.22	0.23	1.35(0.81-2.25)	0.96	0.34
**Random effects**					
Intercept	0.06	0.24		0.25	0.41

Nervous system ICC=4.71%

**Covariates**	**Estimated**	**Standard error**	**Odds ratio（95%CI）**	**T-value**	**P-value**
**Fixed effects**					
Intercept	-5.65	0.34		-16.82	<.0001
Take supplements					
None	Ref	Ref	Ref	Ref	Ref
Taking folic acid only	-0.29	0.30	0.75(0.42-1.35)	-0.96	0.34
Taking multivitamin only	-0.15	0.36	0.86(0.43-1.73)	-0.43	0.67
Taking folic acid plus multivitamins	-0.05	0.29	0.95(0.54-1.68)	-0.17	0.87
**Random effects**					
Intercept	0.15	0.39		0.38	0.36

Genitourinary system ICC=10.23%

**Covariates**	**Estimated**	**Standard error**	**Odds ratio （95%CI）**	**T-value**	**P-value**
**Fixed effects**					
Intercept	-6.56	0.47		-13.94	<.0001
Take supplements					
None	Ref	Ref	Ref	Ref	Ref
Taking folic acid only	0.68	0.40	1.97(0.90-4.30)	1.70	0.09
Taking multivitamin only	1.17	0.42	3.22(1.42-7.29)	2.80	0.01
Taking folic acid plus multivitamins	0.68	0.39	1.98(0.91-4.30)	1.73	0.08
**Random effects**					
Intercept	0.34	0.59		0.58	0.30

Chromosome malformation ICC=6.12%

**Covariates**	**Estimated**	**Standard error**	**Odds ratio (95%CI)**	**T-value**	**P-value**
Fixed effects					
**Intercept**	-6.25	0.42		-14.85	<.0001
Take supplements					
None	Ref	Ref	Ref	Ref	Ref
Taking folic acid only	0.61	0.38	1.85(0.87-3.90)	1.61	0.11
Taking multivitamin only	0.95	0.41	2.57(1.16-5.73)	2.32	0.02
Taking folic acid plus multivitamins	0.66	0.38	1.93(0.92-4.04)	1.74	0.08
**Random effects**					
Intercept	0.19	0.43		0.44	0.34

Ear, face and neck ICC=19.21%

**Covariates**	**Estimated**	**Standard error**	**Odds ratio （95%CI）**	**T-value**	**P-value**
**Fixed effects**					
Intercept	-6.30	0.50		-12.48	<.0001
Take supplements					
None	Ref	Ref	Ref	Ref	Ref
Taking folic acid only	-0.18	0.31	0.84(0.46-1.54)	-0.57	0.57
Taking multivitamin only	-0.03	0.35	0.97(0.48-1.94)	-0.09	0.93
Taking folic acid plus multivitamins	-0.27	0.30	0.76(0.42-1.39)	-0.89	0.37
**Random effects**					
Intercept	0.80	0.89		0.90	0.21

Oral clefts ICC=0.13%

**Covariates**	**Estimated**	**Standard error**	**Odds ratio (95%CI)**	**T-value**	**P-value**
**Fixed effects**					
Intercept	-6.92	0.58		-11.88	<0.001
Take supplements					
None	Ref	Ref	Ref	Ref	Ref
Taking folic acid only	0.21	0.60	1.23(0.38-4.00)	0.35	0.73
Taking multivitamin only	0.39	0.69	1.48(0.38-5.67)	0.57	0.57
Taking folic acid plus multivitamins	0.50	0.59	1.64(0.52-5.23)	0.84	0.40
**Random effects**					
Intercept	-	-		-	-

Limb ICC=3.62%

Covariates	Estimated	Standard error	Odds ratio (95%CI)	T-value	P-value
**Fixed effects**					
Intercept	-5.83	0.35		-16.46	< 0.001
Take supplements					
None	Ref	Ref	Ref	Ref	Ref
Taking folic acid only	-0.07	0.33	0.93(0.49-1.79)	-0.21	0.84
Taking multivitamin only	0.68	0.36	1.97(0.97-4.00)	1.87	0.06
Taking folic acid plus multivitamins	-0.03	0.33	0.97(0.51-1.84)	-0.09	0.93
**Random effects**					
Intercept	0.11	0.34		0.32	0.38
