# Supplementary material for: Is the Tradeoff between Folic Acid or/and Multivitamin Supplementation against Birth Defects in Early Pregnancy Reconsidered? Evidence Based on a Chinese Birth Cohort Study
Source: Nutrients. 2023 Jan 5;15(2):279. doi: 10.3390/nu15020279 (PMC9865336; doi:10.3390/nu15020279)
Supplement: Supplementary file 1 [file nutrients-15-00279-s001.zip › Table S2.pdf]

Table S2. Incidence of Gestational Diabetes Mellitus among different exposure groups.

Diseases	Total (120 652)		Exposure of only FA (40 204)		Exposure of only MV (5567)		Exposure of FA and MV (71 538)		Without exposure of FA and MV (3343)	
	n	Incidence per 1000	n	Incidence per 1000	n	Incidence per 1000	n	Incidence per 1000	n	Incidence per 1000
Gestational Diabetes Mellitus	2081	17.2 (16.5-18.0)	586	14.6 (13.4-15.8)	103	18.5 (15.1-22.4)	1359	19.0 (18.0-20.0)	33	9.9 (6.8-13.8)
