# Supplementary figures and images for: Is the Tradeoff between Folic Acid or/and Multivitamin Supplementation against Birth Defects in Early Pregnancy Reconsidered? Evidence Based on a Chinese Birth Cohort Study
Source: Nutrients. 2023 Jan 5;15(2):279. doi: 10.3390/nu15020279 (PMC9865336; doi:10.3390/nu15020279)

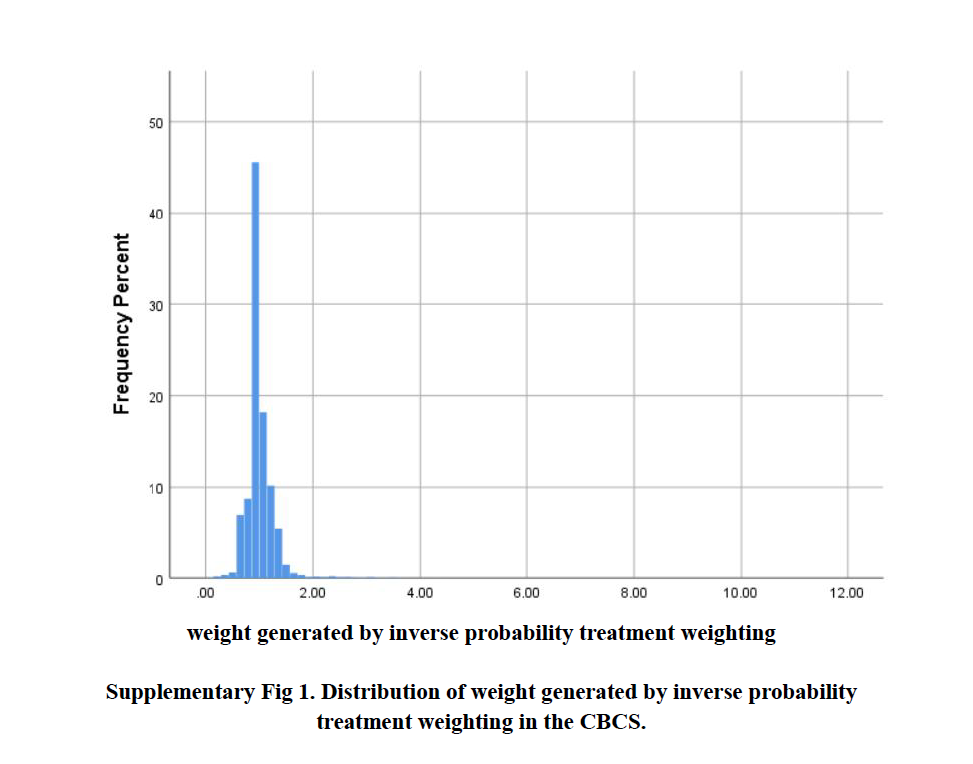

Supplement: Supplementary file 1 [file nutrients-15-00279-s001.zip › Figure S1.tif]
